# First Confirmed Cases of Middle East Respiratory Syndrome Coronavirus (MERS-CoV) Infection in the United States, Updated Information on the Epidemiology of MERS-CoV Infection, and Guidance for the Public, Clinicians, and Public Health Authorities — May 2014

**Published:** 2014-05-16

**Authors:** Stephanie R. Bialek, Donna Allen, Francisco Alvarado-Ramy, Ray Arthur, Arunmozhi Balajee, David Bell, Susan Best, Carina Blackmore, Lucy Breakwell, Andrew Cannons, Clive Brown, Martin Cetron, Nora Chea, Christina Chommanard, Nicole Cohen, Craig Conover, Antonio Crespo, Jeanean Creviston, Aaron T. Curns, Rebecca Dahl, Stephanie Dearth, Alfred DeMaria, Fred Echols, Dean D. Erdman, Daniel Feikin, Mabel Frias, Susan I. Gerber, Reena Gulati, Christa Hale, Lia M. Haynes, Lea Heberlein-Larson, Kelly Holton, Kashef Ijaz, Minal Kapoor, Katrin Kohl, David T. Kuhar, Alan M. Kumar, Marianne Kundich, Susan Lippold, Lixia Liu, Judith C. Lovchik, Larry Madoff, Sandra Martell, Sarah Matthews, Jessica Moore, Linda R. Murray, Shauna Onofrey, Mark A. Pallansch, Nicki Pesik, Huong Pham, Satish Pillai, Pam Pontones, Sarah Poser, Kimberly Pringle, Scott Pritchard, Sonja Rasmussen, Shawn Richards, Michelle Sandoval, Eileen Schneider, Anne Schuchat, Kristine Sheedy, Kevin Sherin, David L. Swerdlow, Jordan W. Tappero, Michael O. Vernon, Sharon Watkins, John Watson

**Affiliations:** 1 Division of Viral Diseases, National Center for Immunization and Respiratory Diseases, CDC; 2 Indiana State Department of Health; 3 Division of Global Migration and Quarantine, National Center for Emerging and Zoonotic Infectious Diseases, CDC; 4 Division of Global Health Protection, Center for Global Health, CDC; 5 Lake County Health Department, Indiana; 6 Florida Department of Health; 7 Epidemic Intelligence Service, Division of Scientific Education and Professional Development, CDC; 8 Division of Bacterial Diseases, National Center for Immunization and Respiratory Diseases, CDC; 9 Division of Healthcare Quality Promotion, National Center for Emerging and Zoonotic Infectious Diseases, CDC; 10 Illinois Department of Public Health; 11 Dr. P. Phillips Hospital, Orlando, Florida; 12 Massachusetts Department of Public Health; 13 Cook County Department of Public Health, Illinois; 14 Community Hospital, Munster, Indiana; 15 Florida Department of Health-Orange County; 16 Division of Preparedness and Emerging Infections, National Center for Emerging and Zoonotic Infectious Diseases, CDC; 17 Influenza Coordination Unit, Office of Infectious Diseases, CDC; 18 National Center for Chronic Disease Prevention and Health Promotion, CDC; 19 Office of the Director, National Center for Immunization and Respiratory Diseases, CDC

Since mid-March 2014, the frequency with which cases of Middle East respiratory syndrome coronavirus (MERS-CoV) infection have been reported has increased, with the majority of recent cases reported from Saudi Arabia and United Arab Emirates (UAE). In addition, the frequency with which travel-associated MERS cases have been reported and the number of countries that have reported them to the World Health Organization (WHO) have also increased. The first case of MERS in the United States, identified in a traveler recently returned from Saudi Arabia, was reported to CDC by the Indiana State Department of Health on May 1, 2014, and confirmed by CDC on May 2. A second imported case of MERS in the United States, identified in a traveler from Saudi Arabia having no connection with the first case, was reported to CDC by the Florida Department of Health on May 11, 2014. The purpose of this report is to alert clinicians, health officials, and others to increase awareness of the need to consider MERS-CoV infection in persons who have recently traveled from countries in or near the Arabian Peninsula.* This report summarizes recent epidemiologic information, provides preliminary descriptions of the cases reported from Indiana and Florida, and updates CDC guidance about patient evaluation, home care and isolation, specimen collection, and travel as of May 13, 2014.

MERS-CoV was first reported to cause human infection in September 2012. Since mid-March 2014, the frequency with which cases have been reported has increased.† As of May 12, 2014, 536 laboratory-confirmed§ cases of MERS-CoV infection have been reported by WHO (Figure 1). This includes 145 deaths. All reported cases have been directly or indirectly linked through travel or residence to seven countries: Saudi Arabia, UAE, Qatar, Oman, Jordan, Kuwait, and Yemen (Figure 2). Public health investigations are ongoing to determine the reason for the increase in cases.

The median age of persons with laboratory-confirmed MERS-CoV infection is 49 years (range = <1–94 years); 346 (65%) cases are in males, and 104 (19%) occurred in health-care workers. Although 62% of cases involved severe respiratory illness requiring hospitalization, 32 (5%) occurred in persons who had mild symptoms or illness not requiring hospitalization and 110 (21%) were asymptomatic, generally as a result of contact investigations.

Countries outside the Arabian Peninsula with travel-associated MERS cases reported by WHO include the United Kingdom (UK), France, Tunisia, and Italy, where cases were reported in 2013(1–4), and Malaysia, Greece, Egypt, and the United States, where cases have been reported in 2014 (Figure 2). The travel-associated MERS cases reported by countries outside the Arabian Peninsula in 2014 occurred in persons with residence in or travel to Saudi Arabia. In addition, cases have occurred among travelers from Saudi Arabia to UAE and Jordan. Malaysia reported a case on April 17, 2014, in a man aged 54 years with underlying health problems. He had traveled to Jeddah, Saudi Arabia, visited a camel farm and consumed camel milk during his trip. He sought treatment in Malaysia on April 7 and died on April 13. Greece reported a case on April 18, 2014, in a male Greek citizen aged 69 years residing in Jeddah, Saudi Arabia, who traveled to Greece on April 17. His source of infection remains unclear. During the 14 days before onset of illness, he had extensive contact with a family member who was hospitalized in Jeddah but not with MERS-CoV infection. Egypt reported a case in a male aged 27 years who had been living in Riyadh, Saudi Arabia, for the past 4 years and returned to Egypt on April 25. He had contact with two persons with laboratory-confirmed MERS-CoV infection in Saudi Arabia. The UAE Ministry of Health reported a case on March 30, 2014, in a male aged 64 years who had traveled to Saudi Arabia, where he visited a camel farm. A case of MERS was reported by Jordan on April 22, 2014, in a male aged 25 years from Saudi Arabia. He had a family member in Saudi Arabia who was previously reported by WHO as having MERS.

The first case of MERS in the United States was reported to CDC by the Indiana State Department of Health on May 1, 2014, and confirmed by CDC on May 2. The case involved a male U.S. citizen aged ≥60 years who lived and worked in Saudi Arabia in a hospital in which patients with MERS had received care. He began feeling unwell on or around April 18 with a low-grade fever and myalgia without any respiratory symptoms. He traveled by commercial airlines from Saudi Arabia to Chicago, Illinois, on April 24, 2014, and then traveled by bus from Chicago to his destination in Indiana. On April 27, he developed shortness of breath, nonproductive cough, increasing fever, and rhinorrhea. On April 28, he was evaluated at and admitted to a hospital in Indiana. A chest radiograph revealed a right lower lobe infiltrate; chest computed tomography scan revealed bilateral lung infiltrates. The patient required supplemental oxygen, but did not require mechanical ventilation. On May 9, the patient was no longer symptomatic and health officials verified that the patient had tested negative for MERS-CoV by polymerase chain reaction (PCR) in two sets of sputum, nasopharyngeal/oropharyngeal, and serum specimens collected on different days; the patient was considered to be fully recovered and was discharged from the hospital.

Before implementation of contact and airborne infection control precautions at the hospital in Indiana, 53 health-care personnel (HCP) had contact with the patient. Household contacts (who were assumed to be exposed), a community contact (a business associate in Cook County, Illinois, with whom the patient had extended face-to-face contact on April 25), and exposed HCP were asked to monitor themselves twice daily for symptoms and fever for 14 days after exposure, the period in which symptoms of MERS would be expected to appear. Household contacts and exposed HCP were recommended to wear a mask when outside of the house or in contact with other household members while on voluntary home quarantine¶ for 14 days after contact. HCP who had unprotected close contact with the patient and were asymptomatic returned to work 14 days after the last exposure and confirmed negative laboratory results for MERS-CoV. Nasopharyngeal and serum specimens collected from all household, community, and HCP contacts have tested negative by PCR for MERS-CoV.

The Indiana case involved a person who traveled on commercial flights between Saudi Arabia and the UK and between the UK and Chicago while he was symptomatic and potentially contagious. He then traveled for 70 minutes by bus from Chicago to his final destination in Indiana. For the two flights, the UK has jurisdiction for the flight from Saudi Arabia to the UK and the United States has jurisdiction for the flight from the UK to Chicago. Because little is known about the modes of transmission of MERS-CoV, CDC included all passengers and crew aboard the flight from the UK to Chicago and the bus in a contact investigation. Eighty airline passengers (including two who were also on the Saudi Arabia to UK flight) and 12 crew members were identified for follow-up from the flight between the UK and Chicago. As of May 12, 2014, a total of 58 airline passengers on the flight from the UK to Chicago have been contacted by CDC or state and local health departments; health authorities in other countries were notified about the other 22 passengers. Eight passengers on the Saudi Arabia to UK flight who later traveled to the United States have also been contacted. Four airline passengers on the flight from the UK to Chicago reported mild respiratory symptoms. Although these symptoms did not meet the case definition for a patient under investigation for MERS, to be especially cautious given the limited data on transmission of MERS-CoV, CDC and state health departments closely monitored the status of these four passengers for the duration of the 14 day incubation period. All airline crew were contacted and reported no symptoms. Nine passengers and a driver were on the bus that the affected person traveled on from Chicago to his final destination in Indiana. Five bus passengers and the bus driver were contacted and reported no illness. All airline and bus contacts were asked to monitor their body temperature twice daily and to report any fever (temperature of 100°F [37.8°C] or higher) or respiratory symptoms to their state or local health department until 14 days after the flight or bus trip.

A second imported case of MERS in the United States, identified in a traveler, was reported to CDC by the Florida Department of Health on May 11, 2014, and confirmed by CDC on May 11. The traveler, a health-care provider aged ≥40 years who resides and works in Saudi Arabia, is not linked to the case confirmed in Indiana. On May 1, the patient traveled by commercial airline from Saudi Arabia to the UK, the UK to Boston, Massachusetts; then Boston to Atlanta, Georgia; and then Atlanta to Orlando, Florida. The patient began feeling unwell during the flight from Saudi Arabia to the UK and continued to feel unwell on subsequent flights, with symptoms including myalgia, fever, chills, and a slight cough. He continued to have intermittent fevers, nausea, and severe myalgia during his time in Orlando, and on May 9, he went to a hospital emergency department. He was admitted to that hospital the same day to be evaluated for an acute viral syndrome. At the time of admission, the patient was afebrile. Public health and hospital officials have implemented infection control precautions (standard, contact, and airborne) at the hospital and are interviewing HCP who had close contact** with the patient and as well as household contacts to obtain detailed information on their exposures and monitor their health. CDC and state and local health departments are conducting airline contact tracing to identify and notify U.S. travelers who might have been exposed to this infected traveler.

CDC used BioMosaic†† to analyze International Air Transport Association travel volume data for May and June from Saudi Arabia and UAE to North America for 2010–2012. This analysis showed that Cook County, which includes Chicago O’Hare airport, historically has the fourth highest volume of arriving travelers from Saudi Arabia and UAE for the months of May and June (Figure 3). Five cities in the United States accounted for 75% of arrivals from Saudi Arabia and UAE; approximately 100,000 travelers are estimated to arrive in these five cities from Saudi Arabia and UAE in May and June 2014.

## Discussion

This report describes the first two cases of MERS identified in the United States. These cases highlight the critical role that health-care providers play in considering a diagnosis of MERS-CoV infection in persons who develop respiratory symptoms within 14 days after traveling from countries in or near the Arabian Peninsula. Recent travelers might seek medical care distant from cities served by international air connections and all HCP need to be vigilant, including those outside of cities with the highest number of arriving travelers from the Arabian Peninsula. Health-care providers and health departments throughout the United States should be prepared to consider, detect, and manage cases of MERS.

Recommendations might change as additional data become available. Guidance on evaluation of patients for MERS, infection control, interim home care and isolation, and collection and testing of clinical specimens for MERS-CoV infection is summarized below and is available on CDC’s MERS website ( http://www.cdc.gov/coronavirus/mers/index.html). No specific treatment for MERS-CoV infection is currently available. WHO has posted guidance for clinical management of MERS patients at http://www.who.int/csr/disease/coronavirus_infections/InterimGuidance_ClinicalManagement_NovelCoronavirus_11Feb13u.pdf?ua=1.

### Evaluating patients

CDC’s Interim Guidance for Health Professionals was updated on May 9, 2014, to include additional guidance on evaluation of patients and close contacts. Health-care professionals should evaluate for MERS-CoV infection in patients in the United States who meet the following criteria: A) fever and pneumonia or acute respiratory distress syndrome (based on clinical or radiologic evidence) and either 1) a history of travel from countries in or near the Arabian Peninsula within 14 days before symptom onset or 2) close contact with a symptomatic traveler who developed fever and acute respiratory illness (not necessarily pneumonia) within 14 days after traveling from countries in or near the Arabian Peninsula, or 3) is a member of a cluster of patients with severe acute respiratory illness (e.g., fever and pneumonia requiring hospitalization) of unknown etiology in which MERS is being evaluated in consultation with a state or local health department; or B) close contact with a confirmed or probable case of MERS while the affected person was ill. Additional guidance for health-care providers is available at http://www.cdc.gov/coronavirus/mers/interim-guidance.html. Health-care providers should immediately report any person being evaluated for MERS-CoV infection who meets the criteria of a patient under investigation to their state or local health department. States will then notify CDC. Case definitions are available at http://www.cdc.gov/coronavirus/mers/case-def.html. Health-care providers should contact their state or local health department if they have any questions.

### Infection control

HCP should adhere to recommended infection-control measures, including standard, contact, and airborne precautions (including eye protection), while managing symptomatic contacts and patients who are patients under investigation or who have probable or confirmed MERS-CoV infections. Additional CDC guidance on MERS-CoV infection control in health-care settings is available at http://www.cdc.gov/coronavirus/mers/infection-prevention-control.html.

### Interim home care and isolation

Ill persons who are being evaluated for MERS and do not require hospitalization for medical reasons may be cared for and isolated in their home. Health-care providers should contact their state or local health department to determine whether home isolation or additional measures are indicated because recommendations might be modified as more data become available. Isolation is defined as the separation or restriction of activities of an ill person with a contagious disease from those who are well. Additional information on home care and isolation guidance is available at http://www.cdc.gov/coronavirus/mers/hcp/home-care.html.

### Collecting and testing clinical specimens for MERS-CoV infection

To increase the likelihood of detecting MERS-CoV infection, CDC recommends collecting multiple specimens from different sites at different times after symptom onset. For suspected MERS cases, health-care providers should collect the following specimens for submission to CDC or the appropriate public health laboratory: nasopharyngeal swab, oropharyngeal swab (which can be placed in the same tube of viral transport medium as nasopharyngeal swabs), sputum, serum, and stool/rectal swab. In addition to nasopharyngeal/oropharyngeal specimens, collection of lower respiratory specimens (e.g., sputum or bronchoalveolar lavage) is recommended because MERS-CoV infection has been confirmed in sputum of patients who tested negative by PCR for MERS-CoV in nasopharyngeal/oropharyngeal specimens. Personnel collecting specimens should wear recommended personal protective equipment (i.e., gloves, gowns, eye protection, and respiratory protection), and recommended infection control precautions should be used when collecting specimens. Health-care providers should notify their state or local health departments if they suspect MERS-CoV infection in a person. State or local health departments should notify CDC of patients under investigation and any positive MERS-CoV test. Additional information is available at http://www.cdc.gov/coronavirus/mers/guidelines-clinical-specimens.html.

### Travel guidance

In response to the recent increase in cases of MERS in countries in and near the Arabian Peninsula, CDC updated its advice for travelers. CDC’s travel notice has been upgraded to a Level 2 Alert,§§ which includes enhanced precautions for travelers to countries in or near the Arabian Peninsula who plan to work in health-care settings. These travelers should review CDC’s recommendations for infection control for confirmed or suspected MERS patients before they depart, practice these precautions while in the area, and monitor their health closely during and after their travel.

CDC continues to recommend that all U.S. travelers to countries in or near the Arabian Peninsula protect themselves from respiratory diseases, including MERS, by washing their hands often and avoiding contact with persons who are ill. If travelers to the region have onset of fever with cough or shortness of breath during their trip or within 14 days of returning to the United States, they should seek medical care. They should call ahead to their health-care provider and mention their recent travel so that appropriate isolation measures can be taken in the health-care setting.

More detailed travel recommendations related to MERS are available at http://wwwnc.cdc.gov/travel/notices/alert/coronavirus-arabian-peninsula-uk. In addition to the Travelers’ Health website, CDC is using partner distribution lists, e-mail subscription channels, social media, and airport messages to alert U.S. travelers and clinicians about precautions for MERS.

## Figures and Tables

**FIGURE 1:**
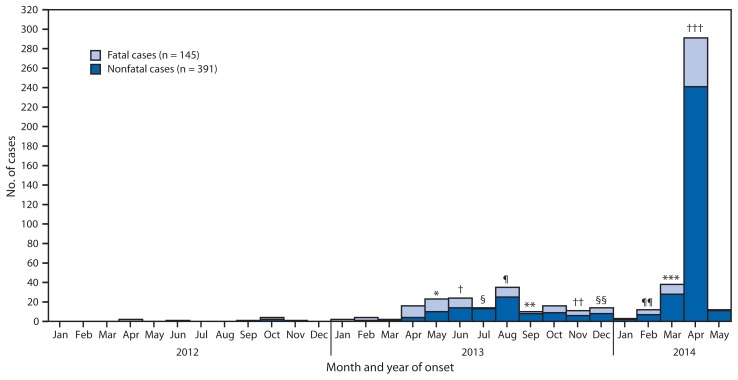
Number of confirmed cases of Middle East respiratory syndrome coronavirus infection (145 fatal and 391 nonfatal) reported by the World Health Organization (WHO) as of May 12, 2014, by month of illness onset — worldwide, 2012–2014 * Case count for May assumes that three cases included in WHO announcements on May 22, 23, and June 2, 2013, had symptom onset during May 2013. ^†^ Case count for June assumes that 22 cases included in WHO announcements on June 14, 17, 22, 23, 26, and July 5, 7, 11, 2013, had symptom onset during June 2013. ^§^ Case count for July assumes that 10 cases included in WHO announcements on July 18, July 21, and August 1, 2013 had symptom onset during July 2013. ^¶^ Case count for August assumes that 25 cases included in WHO announcements (six cases on August 28, one case August 29, two cases August 30, and 16 cases September 16) had symptom onset during August 2013. ** Case count for September assumes that four cases included in WHO on October 4 and 24 announcements had symptom onset during September 2013. ^††^ Assumes that three cases had symptom onset during November 2013. ^§§^ Assumes that six cases had symptom onset during December 2013. ^¶¶^ Assumes that two cases had symptom onset during February 2014. *** Assumes that 16 cases had symptom onset during March 2014. ^†††^ Assumes that 66 cases had symptom onset during April 2014.

**FIGURE 2:**
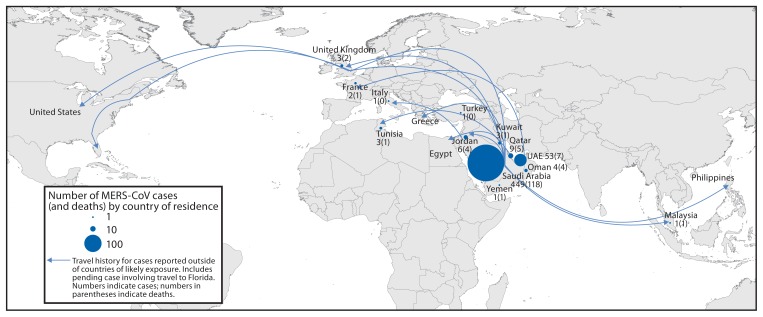
Confirmed cases of Middle East respiratory syndrome coronavirus (MERS-CoV) infection (N = 536) (and deaths) reported by the World Health Organization as of May 12, 2014, and history of travel from in or near the Arabian Peninsula within 14 days of illness onset — worldwide, 2012–2014 **Source:** Adapted from Epidemiological update: Middle East respiratory syndrome coronavirus. Stockholm, Sweden: European Centre for Disease Prevention and Control; 2014. Available at http://www.ecdc.europa.eu/en/press/news/_layouts/forms/News_DispForm.aspx?List=8db7286c-fe2d-476c-9133-18ff4cb1b568&ID=998.

**FIGURE 3:**
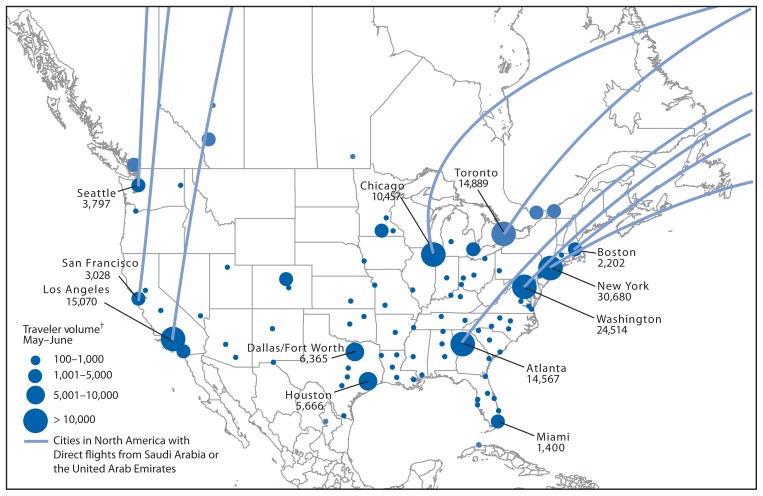
Points of entry and volume of travelers on flights to the United States and Canada from Saudi Arabia and the United Arab Emirates — May–June 2014* **Source:** BioMosaic, an analytic tool for integrating demography, migration, and health data developed in collaboration between the University of Toronto, Boston Children’s Hospital, and CDC’s Division of Global Migration and Quarantine. * Excludes cities with fewer than 100 travelers from affected areas. ^†^ Based on total number of arrivals at final destination in North America.

## Footnotes

* Countries considered in or near the Arabian Peninsula include Bahrain, Iraq, Iran, Israel, Jordan, Kuwait, Lebanon, Oman, Palestinian Territories, Qatar, Saudi Arabia, Syria, UAE, and Yemen.

† Additional information available at http://www.who.int/csr/disease/coronavirus_infections/archive_updates/en.

§ Confirmatory laboratory testing requires a positive PCR on at least two specific genomic targets for MERS-CoV or a single positive target with sequencing on a second.

¶ Quarantine is defined as the separation or restriction of movement of well persons who might have been exposed to a communicable disease, while determining if they become ill.

** Close contact is defined as 1) any person who provided care for the patient, including a health-care worker or family member, or had similarly close physical contact; or 2) any person who stayed at the same place (e.g. lived with, visited) as the patient while the patient was ill.

†† An analytic tool for integrating demography, migration, and health data developed as collaboration between the University of Toronto (Kamran Kahn), Boston Children’s Hospital (John Brownstein), and CDC’s Division of Global Migration and Quarantine (Martin Cetron).

§§ A Level 2 Alert includes recommendations for travelers to follow new or enhanced precautions for the affected destination because of the increased health risk. Additional information available at http://wwwnc.cdc.gov/travel/notices#travel-notice-definitions.
