# Erratum

**Published:** 2014-06-27

## Vol. 63, No. 19

In the report “First Confirmed Cases of Middle East Respiratory Syndrome Coronavirus (MERS-CoV) Infection in the United States, Updated Information on the Epidemiology of MERS-CoV Infection, and Guidance for the Public, Clinicians, and Public Health Authorities — May 2014,” the arrow in Figure 2 showing travel from Saudi Arabia to the Philippines should instead show travel from the United Arab Emirates (UAE) to the Philippines.

